# Variants of *IL6*, *IL10*, *FCN2*, *RNASE3*, *IL12B* and *IL17B* loci are associated with *Schistosoma mansoni* worm burden in the Albert Nile region of Uganda

**DOI:** 10.1371/journal.pntd.0011796

**Published:** 2023-11-30

**Authors:** Oscar Asanya Nyangiri, Julius Mulindwa, Joyce Namulondo, Anna Kitibwa, Jacent Nassuuna, Alison Elliott, Magambo Phillip Kimuda, Alex Boobo, Barbara Nerima, Moses Adriko, Nathan J. Dunton, Gaganjit Kaur Madhan, Mark Kristiansen, Miriam Casacuberta-Partal, Harry Noyes, Enock Matovu, on behalf of the TrypanoGEN+ Research group of the H3Africa consortium

**Affiliations:** 1 Department of Biotechnical and Diagnostic Sciences, College of Veterinary Medicine Animal Resources and Biosecurity, Makerere University, Kampala, Uganda; 2 Department of Biochemistry and Sports Sciences, College of Natural Sciences, Makerere University, Kampala, Uganda; 3 Medical Research Council/Uganda Virus Research Institute and London School of Hygiene & Tropical Medicine Uganda Research Unit, Entebbe, Uganda; 4 Vector Borne & NTD Control Division, Ministry of Health, Uganda; 5 UCL Genomics core facility, University College London, London, United Kingdom; 6 Department of Parasitology, Leiden University Medical Center, Leiden, the Netherlands; 7 Centre for Genomic Research, University of Liverpool, Liverpool, United Kingdom; George Washington University, UNITED STATES

## Abstract

**Background:**

Individuals genetically susceptible to high schistosomiasis worm burden may contribute disproportionately to transmission and could be prioritized for control. Identifying genes involved may guide development of therapy.

**Methodology/Principal findings:**

A cohort of 606 children aged 10–15 years were recruited in the Albert Nile region of Uganda and assessed for *Schistosoma mansoni* worm burden using the Up-Converting Particle Lateral Flow (UCP-LF) test detecting circulating anodic antigen (CAA), point-of-care Circulating Cathodic Antigen (POC-CCA) and Kato-Katz tests. Whole genome genotyping was conducted on 326 children comprising the top and bottom 25% of worm burden. Linear models were fitted to identify variants associated with worm burden in preselected candidate genes. Expression quantitative trait locus (eQTL) analysis was conducted for candidate genes with UCP-LF worm burden included as a covariate. Single Nucleotide Polymorphism loci associated with UCP-LF CAA included *IL6* rs2066992 (OR = 0.43, *p* = 0.0006) and rs7793163 (OR = 2.0, *p* = 0.0007); *IL21* SNP kgp513476 (OR 1.79, p = 0.0025) and *IL17B* SNP kgp708159 (OR = 0.35, p = 0.0028). A haplotype in the *IL10* locus was associated with lower worm burden (OR = 0.53, p = 0.015) and overlapped SNPs rs1800896, rs1800871 and rs1800872. Significant haplotypes (p<0.05, overlapping significant SNP) associated with worm burden were observed in *IL6* and the Th17 pathway *IL12B* and *IL17B* genes. There were significant eQTL in the *IL6*, *IL5*, *IL21*, *IL25* and *IFNG* regions.

**Conclusions:**

Variants associated with *S*. *mansoni* worm burden were in *IL6*, *FCN2*, *RNASE3*, *IL10*, *IL12B* and *IL17B* gene loci. However only eQTL associations remained significant after Bonferroni correction. In summary, immune balance, pathogen recognition and Th17 pathways may play a role in modulating *Schistosoma* worm burden. Individuals carrying risk variants may be targeted first in allocation of control efforts to reduce the burden of schistosomiasis in the community.

## Author summary

This study investigated genetic risk factors for high worm burden in a cohort of 606 children aged 10–15 years old in the Albert Nile region of Uganda. Genotyping was conducted on 326 children who comprised the top and bottom 25% of worm burden. Results showed single nucleotide polymorphisms (SNPs) associated with worm burden in the *IL6*, *IL21 and IL17B* genes, and a haplotype in the *IL10* locus associated with lower worm burden. Expression quantitative trait locus (eQTL) analysis revealed significant gene and worm burden associations in the IL6 region, and expression associations in the *IL5*, *IL21*, *IL25* and *IFNG* loci. These findings suggest that immune balance, pathogen recognition and Th17 pathways may be involved in modulating Schistosoma worm burden and that individuals carrying risk variants may be prioritized to improve control efforts.

## Introduction

Schistosomiasis affects more than 250 million people globally, but Africa bears the highest burden with approximately 80% of cases [1]. The disease is maintained in endemic communities by schistosome eggs shed into water from infected individuals. Epidemiological studies have shown that approximately 20% of individuals maintain the transmission cycle by contributing approximately 80% of transmission [2]. These are likely to be high worm burden individuals who should be monitored and targeted for control. Worm burden resolves to low levels after puberty, and therefore school aged children (SAC) between 5 and 15 years are the primary carriers of high worm burdens [3]. The World Health Organization (WHO) therefore gives a central role to mass drug administration (MDA) using praziquantel especially in SAC as part of the strategy for elimination of schistosomiasis as a public health problem globally by 2030 [4].

Despite MDA, hotspots of transmission persist, and this could be due to a combination of lack of sanitation, re-infection, environmental factors and infection intensity [5]. Host immune factors controlling infection intensity, also referred to as worm burden, may be targeted to increase the efficacy of MDA but they are incompletely understood. Current understanding of schistosomiasis infection associated immunology is complex and has been reviewed [6]. Briefly, different immune components are activated at various stages of schistosome infection and pathology development. Innate and Th1 responses predominate when schistosome larvae penetrate the skin and migrate, while alarmins are released when host barrier cells are damaged [7]. Th2 responses predominate about 6 weeks post infection when schistosome eggs are deposited in the intestine, liver or bladder [8]. Parasite derived antigens drive Th2 polarization [9,10]. In long standing helminth infections immune responses are downregulated, allowing the parasite to survive for longer and minimize host tissue damage [6]. Since host genetics underlies the immune presentation, studies of host genetics represent a potent approach to understanding immune components associated with schistosomiasis pathology.

It is well established that there is a genetic component to susceptibility to high worm burden, with the earliest study identifying a cytokine cluster in chromosome 5 which explained approximately 66% of infection intensity in a Brazilian population [11]. Candidate gene studies have found multiple associations with multiple genes in this region [12]. In a recent review of human schistosomiasis genetics studies, we showed that there is paucity of studies on the Th17 pathway, yet Th17 pathway genes are within four of the five quantitative trait loci (QTL) published for schistosomiasis worm burden and fibrosis [12]. We aimed to explore the genetic component to *Schistosoma mansoni* worm burden in Ugandan children [11,13]. The identification of genes within these QTL that regulate schistosomiasis outcomes can also contribute to development of new therapies [12]. It is therefore important to identify these genetic factors predisposing to high *Schistosoma mansoni* worm burden.

In sub-Saharan Africa which includes Uganda; schistosomiasis was the 7^th^ largest contributor of the global total of 4.5 million disability adjusted life years(DALYs) in 2013 [14]. Most of the high infections of schistosomiasis in Uganda are amongst the shoreline communities of Lakes Albert, Victoria and Kyoga, and the Albert Nile [15,16]. In the current study, we aimed to identify genetic factors associated with high worm burden in Ugandan children aged 10–15 years. We conducted a survey in the hot spots along the Albert-Nile shoreline in the district of Pakwach in Northern Uganda where high *S*. *mansoni* worm burdens as measured by POC-CCA and UCP-LF CAA were recorded [16]. This area has historically had high *S*. *mansoni* infection intensity [17–19]. We genotyped genes already associated with schistosomiasis in human schistosomiasis genetic literature (*IFNG*, *IL10*, *IL13*, *IL4*, *IL5*, *STAT6*, *CTLA4*, *FCN2*, *COLEC11*, *ABO*, *RNASE3*), those in schistosomiasis infection QTL for which we could not find candidate gene studies in the literature (*IL17A*, *IL17B*, *IL17F*, *IL6R*, *IL12B*) and some in the Th17 pathway, which is underrepresented in schistosomiasis genetic association studies (*IL1A*, *IL1B*, *TGFB1*, *IL6*, *IL21*, *IL23A*, *IL25*, *IL17RA*), as described before [20].

## Methods

The full protocol for this study was published prior to undertaking the genotyping [20]. Here we briefly summarize the methods.

### Ethics statement

This study was approved by the Makerere University Ethics committee and the Uganda National Council of Science and Technology (UNCST No. HS 118). The study was conducted in the context of the National Control Program by the Vector Borne Disease Control Division of the Ministry of Health in Uganda that has implemented the Program in Pakwach (former Nebbi District) for over 19 years since 2003. The study targeted children 10 to 15 years old. Within the target villages in the Lake Albert region, parents were informed of the study and gave written informed consent on behalf of children under 12 years. In addition, those over 12 years gave their assent to participate in the study. As described before in this same population [16], if a child was observed to have *S*. *mansoni* eggs in their stool, they were offered free treatment, which consisted of praziquantel at a dosage of 40mg/kg administered by trained Ministry of Health personnel, assisted by the district health worker.

### Sample collection in Lake Albert

Sample collection was done as previously described [16].The overall study design is summarized in S1 Fig. Pakwach district is located along the western bank of the Albert Nile (Latitude:2.461944; Longitude:31.498333). Briefly, the Pakwach District Health Officers were informed of the study and District Health Teams identified families in the Lake Albert villages in the eligible age range. These were children 10–15 years. They were recruited from their homes by the District Health Team. A stool sample was collected for Kato-Katz (KK), a urine sample was collected for the qualitative point of care Circulating Cathodic Antigen (CCA) while a blood sample was collected to be used for the plasma Up-Converting Particle Lateral Flow (UCP-LF) Circulating Anodic Antigen (CAA) test and for DNA and RNA extractions.

### Determination of egg and worm burden

The Kato-Katz test was used to identify and quantify *S*. *mansoni* eggs microscopically from stool as described previously [21]. The POC-CCA test (Rapid Medical Diagnostics, Pretoria, South Africa) was used to detect circulating antigen derived from the adult worms. Qualitative determination of circulating cathodic antigen(CCA) was done using a scale of +0.5, +1, +2, +3, +4 as previously described [16]. The quantitative UCP-LF CAA test was conducted on the plasma, which was evaluated for circulating anodic antigens derived from worms as described before [22,23]. UCP-LF CAA is the most sensitive test compared to KK and POC-CCA and shows a correlation with infection intensity as assessed by the conventional KK [22]. It was necessary to capture the correlated measures captured by KK and POC-CCA to assess the validity of the measures from UCP-LF CAA in the region. We confirmed expected correlations in order of sensitivity to capture worm burden as follows, from most sensitive to least sensitive: UCP-LF CAA > POC-CCA > KK in samples from this same population as published before [16]; and therefore the most sensitive measure UCP-LF CAA was retained for further analysis. The SCAA20 UCP-LF assay was used.

### DNA extraction and genotyping

Venous blood samples were collected from the children into EDTA tubes for which 200μl was used for DNA extraction using the Qiagen DNA blood kit (Qiagen, Germany) following the manufacturer’s instructions. The resultant DNA was quantified (Qubit, ThermoFisher Scientific) and approximately 1μg was sent for genotyping on the Illumina H3Africa chip at University College London Genomics Facility (London, UK).

### Rationale for extreme phenotype sampling

Li and colleagues [24] described the advantages of using extreme phenotype sampling. The power to detect association is approximately equal to using the full set of samples while using the most informative samples at the extremes of the phenotypes of interest. In our case worm burden is the phenotype of interest and therefore we genotyped individuals with the top 25% and bottom 25% of worm burden based on the UCP-LF CAA test [20]. Individuals without detectable worms for UCP-LF CAA were excluded since it was not known if they had sterile immunity to infection or had simply not been exposed.

### Candidate gene selection

We reviewed the literature on the genetics of schistosome worm burdens [12] and selected genes that have previously been associated with worm burden or that might plausibly account for quantitative trait loci for worm burden genotyping. The list of candidate genes was included in our published protocol [20]. These are *IFNG*, *IL10*, *IL13*, *IL4*, *IL5*, *STAT6*, *CTLA4*, *FCN2*, *COLEC11*, *ABO*, *RNASE3*, *IL17A*, *IL17B*, *IL17F*, *IL6R*, *IL12B*, *IL1A*, *IL1B*, *TGFB1*, *IL6*, *IL21*, *IL23A*, *IL25*, *IL17RA*. Literature supporting the candidate gene selection includes: *IFNG*[25], *IL10* [26,27], *IL13* [25,28], *IL4* [29], *CTLA4* [30], *FCN2*[31], *COLEC11* [32], *ABO* [33], *RNASE3* [34] and those not genetically investigated before in the literature but which have a biological pathway relevant to schistosomiasis or and are in eQTL regions previously associated with schistosomiasis outcomes including Th17 pathway genes such as *IL12B*, *IL17A*, *IL17B*, *IL25*, *IL17F*, *IL17RA*, *IL1A*, *IL1B*, *TGFB1*, *IL6R*, *IL6*, *IL21*, *IL23A*. We noted the possible role of these genes in our previous publication [12]. All SNP on the genotyping chip within 100kb of the candidate genes were included in the study.

### Genotype calling and quality control

Samples were genotyped on the Illumina H3Africa custom chip [35] at the UCL Genomics core facility, University College London (London, United Kingdom).

Genotypes were called using the Illumina GenomeStudio package and then exported to PLINK format. The human reference genome version used was GRCh37. Quality control was done using standard methods for keeping individuals and SNPs as determined by visualization of results. The thresholds for exclusion were individual missingness > 0.025, genotype missingness > 0.05, minor allele frequency < 0.05, Hardy Weinberg equilibrium (*p* value < 0.001), sex check and close relatives pi-hat > 0.2 and extreme heterozygosity < 0.256. Principal component analyses were performed using Plink to identify and remove outliers which may have an undue influence on genetic results.

### Use of haplotypes in addition to SNPs

SNPs that have been associated with outcomes of infections on genotyping chips are not often the causal variants [36]. The power to detect association depends on the linkage between marker SNP and causal SNP, and the frequencies of marker and causal SNP. Differences in frequencies between marker and causal SNP will reduce the power. We used haplotypes of linked SNPs in our candidate gene association studies. Haplotypes are expected to more closely reflect causal allele frequencies and increase the number of informative loci.

### Power

Tables and simulations of power in extreme phenotype sampling are described in [24]. In a multiplicative model a 20% fraction from each extreme phenotype would give approximately 90% power to detect a 35% increase in risk for MAF 0.2 with sample size 500 (100 cases and 100 controls genotyped) [24]. Most effects in the classical *S*. *mansoni* susceptibility locus (SM1) QTL have been shown to be co-dominant [11,37] and therefore a multiplicative model is appropriate.

### Haplotype identification

SNPs were phased using Shapeit2 [38], haplotype blocks were identified using the BigLD R package [39] and haplotype alleles were assigned to individuals with a custom Perl script as previously described [40].

### Data analysis

Demographic factors were tested for their associations with UCP-LF CAA antigen using Wilcoxon rank sum test for continuous variables and Pearson’s chi-square test for categorical variables. Linear regression analysis in Plink V1.90 (https://zzz.bwh.harvard.edu/plink/) [41] was used for discovery of associations between SNP and worm burden (as a logarithm of the UCP-LF CAA antigen test result) with age, sex, body mass index (BMI), sample collection site and first 20 principal components as covariates. Age, sex, height for age Z score(HAZ) and nutritional status measures have been shown in the literature to be related to schistosomiasis worm burden [16,42,43] and we have previously reported associations with age in this population. Principal components are widely used in association analyses to control for confounding due to population structure [44,45]. Principal component analysis(PCA) is a dimension reduction method that aims to capture the most important patterns of genetic variation in a dataset by transforming the original variables into a new set of uncorrelated variables called principal components [46]. These principal components are linear combinations of the original variables and are ordered in terms of the amount of variation they explain in the data [47].

The whole genome genotyping data after quality control as described in the genotype calling and quality control section, was used in Plink to calculate the first 20 principal components of the data which were also used as covariates in the regression analysis. Plink cannot process multi-allelic haplotypes so associations with haplotypes were detected using linear regression in R 3.5, with logarithm of UCP-LF CAA as the dependent variable and haplotype alleles, age, sex, BMI and collection site as explanatory variables. The dependent variable UCP-LF CAA was chosen over POC-CCA and Kato-Katz as it is the most sensitive to worm burden levels [22]. Individuals carrying haplotypes which had a frequency of less than 5 were excluded from the analysis of that haplotype locus. Haplotype alleles were treated as factors which is equivalent to an additive test for SNP. We used R (https://www.r-project.org/) version 4 for plotting graphics. Bonferroni correction was used to adjust results for multiple testing. False Discovery Rate(<0.05) was applied on the top p-value per haplotype block.

### RNA sequencing and eQTL analysis

From a subset of participants (44 cases and 20 controls as defined by UCP-LF CAA), we collected peripheral blood in PAXgene Blood RNA (PreAnalytiX, US) tubes and extracted RNA using Trizol (Invitrogen, USA). Participants were selected during the POC-CCA screening in the field. The POC-CCA scoring gave information on the intensity of infection for which Paxgene blood was taken from only those individuals who were to participate in the transcriptome analysis study, that is High (>3+), Low (<1+) and Negative (0). Samples with RNA concentrations of more than 1μg as measured by Qubit (Invitrogen, USA) were sent for sequencing at the Center for Genomics Research at the University of Liverpool. The total RNA was subjected to rRNA removal using the QIAseq FastSelect rRNA HMR kits (Qiagen) (NEB, New England Biolabs) and the libraries constructed using the NEBNext Ultra II Directional RNA Library Prep Kit. On the Illumina NovaSeq S4 (Illumina), the libraries were sequenced in a 2x150 read format with a target depth of 30m read pairs per sample. Using HiSat2 [48], FASTQ reads were aligned to the GRCh38.104 release 84 human genome sequence retrieved from Ensembl [49]. eQTL analysis was done in Plink 1.9 using linear regression with log normalized read counts as the dependent variable and an additive model of inheritance for each SNP within 100Kb and 1Mb of the candidate gene boundaries. Covariates used were worm burden (as logarithm of UCP-LF CAA), age, sex, body mass index (BMI) and sample collection site. P values were adjusted for the number of SNPs in the gene region tested using Bonferroni correction.

### Results

#### General participant characteristics

Epidemiological characteristics of participants are presented in Table 1. Of all covariates tested, sex, age, site and BMI, only site was significantly associated with worm burden UCP-LF CAA values (p<0.001).

**Table 1:** Participant characteristics and association with worm burden.

Characteristic	Bottom 25% CAA, N = 148^1^	Top 25% CAA, N = 147^1^	p-value^2^
**BMI kg/m** ^**2**^ **(IQR)**	16.94(15.65,18.23)	16.42(15.38,17.69)	0.11
**Sex**			0.4
F	77(52%)	70(48%)	
M	71(48%)	77(52%)	
**Age**			0.6
10	45(30%)	34(23%)	
11	21(14%)	28(19%)	
12	24(16%)	24(16%)	
13	30(20%)	27(18%)	
14	18(12%)	19(13%)	
15	10(6.8%)	15(10%)	
**Site**			<0.001
Dei	38(26%)	29(20%)	
Kayonga	34(23%)	20(14%)	
Kivuje	22(15%)	23(16%)	
Nyakagei	14(9.5%)	53(36%)	
Panyigoro	40(27%)	22(15%)	
**KatoKatz: epg(IQR)**	24(0,84)	390(159,906)	<0.001
KatoKatz Missing(NA)	40	33	

^1^Median (interquartile range,IQR); n (%)

^2^Wilcoxon rank sum test; Pearson’s Chi-squared test

Of 148 individuals in the bottom 25% of worm burden by CAA test, 40 had did not have KatoKatz data recorded, while of 147 individuals in the Top 25% of worm burden 33 did not have Kato Katz data recorded. There was a significant difference in the median values of epg as measured by Kato-Katz as categorized by CAA worm burden with median(IQR) for the bottom 25% CAA being 24(0,84) and the top 25% CAA being 390(159,906).KatoKatz definitions of infection intensity according to WHO classification is light infections<99epg and heavy infections being >400 epg [50]. 390epg median approximates the 400 epg cutoff of WHO heavy infections for the top 25% of worm burden.

We selected individuals within the top and bottom 25% of worm burden UCP-LF CAA signals for genotyping. We conducted quality control of SNPs and individuals by filtering using thresholds as defined in methods section. Nine outliers in the principal component analysis (PCA) were dropped from further analysis. **S1 Table** lists the SNPs and individuals included after each quality control filter. After quality control steps for SNPs, we had 297 individuals who were included for SNP and haplotype analysis.

### Cytokine genes harbour SNPs significantly associated with worm burden

In order to determine the SNPs associated with worm burden, we fitted a linear model with log UCP-LF CAA as the dependent variable and genotype, age, sex, site, BMI and the first 20principal components as independent variables. The top SNPs with uncorrected *p*<0.05 are shown in Table 2. Full results of all SNPs from the association analyses are shown in **S2 Table**. However, no SNP remained significant after Bonferroni correction for multiple testing. The Bonferroni threshold was 0.00015 (0.05/332 haplotype blocks listed in S1 Appendix).

**Table 2:** Top significant SNP.

Gene	Chr	Position	SNP identifier	Allele^±^	OR^§^	95% LCL	95% UCL	p	N
IL6	7	22768219	snp-known1524107	A	0.43	0.26	0.69	0.0006	290
IL6	7	22768249	rs2066992	A	0.43	0.26	0.69	0.0006	290
IL6	7	22772260	kgp5213503	A	0.43	0.26	0.70	0.0007	290
IL6	7	22683622	rs7793163	G	2.00	1.35	2.98	0.0007	290
FCN2	9	137784813	kgp2203394	A	0.35	0.19	0.65	0.0010	290
IL6	7	22766246	rs1800796	G	0.46	0.29	0.73	0.0012	290
IL6	7	22757843	kgp5881278	G	0.48	0.30	0.76	0.0020	290
IL6	7	22705474	rs6962836	G	1.78	1.24	2.56	0.0020	290
IL21-AS1	4	123511012	kgp513476	C	1.79	1.23	2.59	0.0025	290
IL21	4	123511012	kgp513476	C	1.79	1.23	2.59	0.0025	290
IL17B	5	148828117	kgp708159	A	0.35	0.18	0.69	0.0028	290
IL17B	5	148828417	kgp4348707	G	0.35	0.18	0.69	0.0028	290
IL6	7	22756046	kgp1880927	G	1.70	1.19	2.42	0.0038	290

N is the number of individuals included in the analysis, see S2 Table

^±^Alleles reported by PLINK are typically minor alleles. Compared to the human reference genome, these may be classified as variants but exceptions exist where they are the major variants. The particular allele showing association is therefore reported here to avoid ambiguity.

^§^Odds ratio interpretation: The odds ratio is for a one log increase in the UCP-LF CAA measure of worm burden. Although samples were selected by whether they were in the top or bottom quartiles of the CCA distribution the phenotype used in the association study was log UCP-LF CAA. In an additive genetic model that we fitted, the response variable was worm burden as measured by UCP-LF CAA and the predictor was the additive effect of the allele tested. Therefore, the final result of the model shows how every allele adds/reduces the odds of a 1-unit increase/decrease in worm burden as measured by log UCP-LF CAA.

P is p-value. The Bonferroni threshold was 0.00015 (0.05/332 haplotype blocks listed in S1 Appendix).

The top SNPs were within 100kb *of IL6*, *FCN2*, *IL21* and *IL17B*.

### Haplotypes associated with worm burden

Haplotypes in candidate genes fitted to the linear model predicting CAA yielded results with p<0.05 as shown in **S3 Table**. In **Table 3**, we highlight haplotypes with a frequency of > 5% (30 out of 600) as those with <5% are considered rare [51]. These included haplotype loci overlapping *IL10*, *RNASE3*, *FCN2*, *TGFB1*. However, these did not remain significant after Bonferroni correction. Haplotype blocks contained several alleles which were not significant after FDR correction. We considered the top p-value allele signal per haplotype block since all alleles in a block are linked. Using this approach, 69 out of 332 haplotype loci showed an FDR<0.05. The FDR for the top signal and corresponding alleles are shown in S1 Appendix. We also noted that some significant haplotypes contained significant SNPs at p<0.05 (**S4 Table**).

**Table 3:** Top haplotypes associated with worm burden.

Target Gene^&^	Other genes in 100kb region*	Locus	OR	LCL	UCL	P	Freq (%)	N
*IL10*	SNORD112, Y_RNA, **IL10**, IL19, IL20,DYRK3, MAPKAPK2	Chr1:206940831–206947167	0.53	0.32	0.88	0.015	0.18	106
*IL1A*	SLC20A1, NT5DC4, CKAP2L, IL1A,IL1B	Chr2:113436181–113451498	0.36	0.17	0.75	0.007	0.06	33
*IL1B*	NT5DC4,CKAP2L, IL1A, **IL1B**, AC079753.4, IL37, AC079753.5	Chr2:113521754–113535395	1.92	1.06	3.49	0.033	0.10	57
*IL21*	**IL21**, IL21-AS1, BBS12, CETN4P	Chr4:123545648–123560826	0.64	0.43	0.97	0.035	0.21	127
*IL5*	IRF1-AS1, IRF1, **IL5**,RAD50,TH2LCRR	Chr5:132452142–132452744	0.51	0.29	0.91	0.022	0.09	53
*IL17B*	GRPEL2,PCYOX1L, RP11-331K21.1, AC012613.2, AFAP1L1, RP11-394O4.3, **IL17B**, MIR143HG, AC131025.8, MIR145, CSNK1A1, CTB-89H12.4,CARMN	Chr5:148675225–148682188	0.68	0.47	0.97	0.035	0.36	213
*IL12B*	UBLCP1,CTB-11I22.1, RNU4ATAC2P, **IL12B**, AC008697.1, AC008703.1	Chr5:158806244–158822645	1.70	1.04	2.77	0.035	0.15	90
*IL6*	AC002480.2, AC073072.5, **IL6**, IL6-AS1, AC073072.6, AC073072.7, TOMM7	Chr7:22709003–22742519	0.64	0.42	0.99	0.044	0.25	148
*FCN2*	COL5A1, AL603650.2, RP11-263F14.3, **FCN2**, FCN1, RP11-447M12.2	Chr9:137749815–137755595	1.56	1.00	2.44	0.049	0.18	104
*RNASE3*	RNASE1, RP11-219E7.3, RP11-84C10.3, RNASE2,**RNASE3**, RN7SL189P, RP11-84C10.1, METTL17	Chr14:21280748–21283042	1.48	1.06	2.07	0.022	0.42	250
*TGFB1*	**TGFB1**,CYP2S1,AXL,HNRNPUL1, CTD-2195B23.3, CCDC97, B9D2, BCKDHA, TMEM91, C19orf69, AC011526.1, EXOSC5,B3GNT8,DMAC2,ERICH4	Chr19:41875919–41885221	1.83	1.16	2.90	0.010	0.16	97
*IL17RA*	GAB4, AC006548.26, CECR7, AC006946.16, **IL17RA**, CECR6, CECR5, CECR5-AS1, CECR1, AC005300.5,TMEM121B,HDHD5-AS1,ADA2.	Chr22:17525584–17542650	2.12	1.04	4.33	0.040	0.06	38

*Genes only included if they contain a SNP and are in the 100kb upstream or downstream of the target gene. LCL = 95% Lower Confidence Limit; UCL = 95% Upper Confidence Limit. ^&^ This is a locus containing this gene at 100kb on either side. Many other genes are encompassed in the 100kb flanking region of the primary target locus as shown in column “Other genes in 100kb region”. The Bonferroni threshold was 0.00015 (0.05/332 haplotype blocks listed in S1 Appendix). Column N is the number of chromosomes containing the haplotype who were included in the analysis.

**Fig 1:**
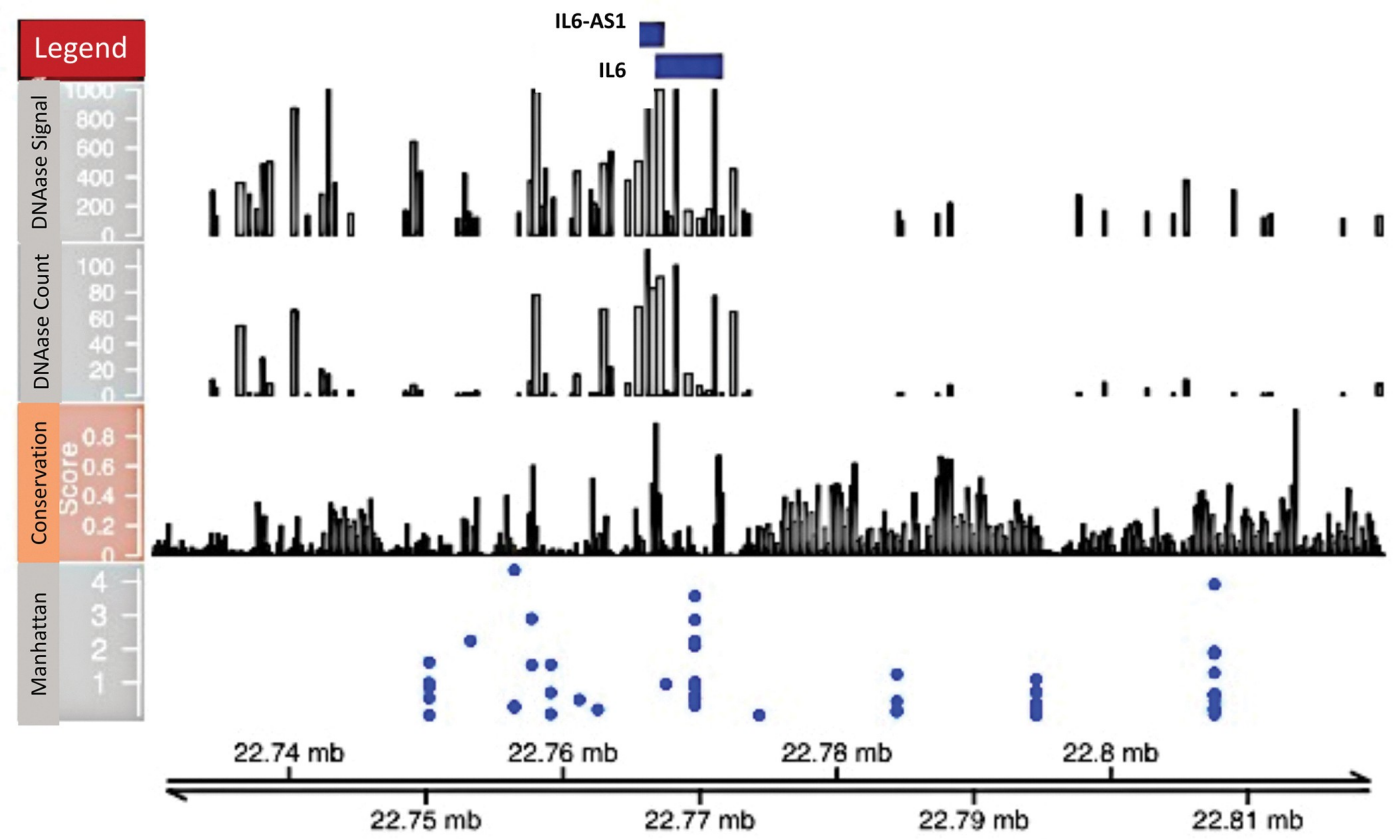
Integrated IL6 locus Manhattan plot, conservation score and DNAse signal. The Manhattan plot was plotted from haplotype p-values in Tables 3, S3 and S4. The strongest Manhattan plot signals are close to regions of high conservation scores and DNAse signals in the IL6 gene locus. Higher conservation scores are associated with conserved regions, where variants are more likely to be functional. High DNAse signal and scores are markers for regulatory and/or transcriptional activity since the chromatin has lost its condensed structure, exposing the DNA and making it accessible for binding of transcription factors.

The IL6 locus showed both haplotypes and SNPs (Fig 1).

### Comparison of results with published studies

Some haplotypes within our candidate genes contain SNPs with published associations with schistosomiasis (Tables 4 and S5). SNPs in literature used for this comparison were previously compiled [12] and are shown in S6 Table.

**Table 4:** Known SNP associations overlapping significant haplotypes for worm burden.

**A: Significant haplotypes overlapping published SNPs**									
**Locus**	**N**	**SNP identifier of published associations**		**Gene**	**OR(CI)**			**P**	**Freq(%)**
Chr12: 57510661–57525756	8	rs324013		STAT6	5.7(1.24–26.26)			0.03	1.35
Chr1:206940831–206947167	106	rs3024495, rs1800871, rs1800872, rs1800896		IL10	0.53(0.32–0.88)			0.02	17.85
**B: Current study effect of haplotypes rs1800871 and rs1800896**									
			**OR (CI)**			**p-value**	**Freq (%)**		
		AT	0.82(0.47–1.42)			0.476	11		
		GT	1.10(0.70–1.74)			0.677	16		
		AC	Reference			NA	62		
		GC	0.52 (0.28–0.97)			0.039	8		

Part A: Known SNPs associations refers to the column “SNP identifier of published associations”. These are contained within the loci named in column “Locus”. The OR(CI), P and Freq(%) are for the haplotype loci. OR(CI) = Odds ratio (95% confidence interval); P = p-value; Freq(%) = Frequency(percentage).

N is number of chromosomes included in the analysis, see N in S5 Table. The Bonferroni threshold was 0.00015 (0.05/332 haplotype blocks listed in S1 Appendix).

### Significant eQTL in IL5 and IL6 loci

We conducted eQTL analysis for the candidate genes. RNA sequence data and covariate data were available for 58 of the samples, comprising 38 infected and 20 uninfected children. Analysis was conducted for 100kb and 1Mb flanking regions respectively. The results are as shown in **Table 5**, for the 100kb analysis (part A) and 1Mb analysis (part B).

**Table 5:** Expression quantitative trait loci (eQTL) associated with worm burden.

**A) 100kb analysis: Flanking regions of 100 kilobases on either side of candidate gene**						
**GENE**	**N**	**Chr**	**Significant SNP identifier**	**BP**	**Raw P**	**Bonferroni Adjusted P**
IL5	101	5	rs201918473	131,821,658	8.02E-05	0.0081
IL5	101	5	kgp11181534	131,822,055	8.02E-05	0.0081
IL5	101	5	kgp7862224	131,822,072	8.02E-05	0.0081
IL5	101	5	kgp6078763	131,819,126	0.00010	0.010
IL5	101	5	rs13165038	131,813,350	0.00010	0.011
IL6	152	7	rs114644824	22,867,134	0.00025	0.038
IL5	101	5	kgp2459073	131,813,219	0.00049	0.049
IL5	101	5	kgp888688	131,819,921	0.00049	0.049
**B) 1Mb analysis: Flanking regions of 1 megabase on either side of candidate gene**						
**GENE**	**N**	**Chr**	**Significant SNP identifier**	**BP**	**Raw P**	**Bonferroni Adjusted P**
IL25	1262	14	h3a_37_14_24685193_T_C	24,685,193	1.30E-05	0.016
IFNG	1353	12	kgp3424793	69,349,042	1.63E-05	0.022
IL21	954	4	kgp10917231	123,831,293	3.74E-05	0.036
IL21	954	4	rs13106595	123,873,534	3.74E-05	0.036
IL5	997	5	rs201918473	131,821,658	8.02E-05	0.080
IL5	997	5	kgp11181534	131,822,055	8.02E-05	0.080
IL5	997	5	kgp7862224	131,822,072	8.02E-05	0.080

N = number of SNP tested for association with gene RNA sequences

The number of individuals who had data analyzed were 38 infected and 20 uninfected children.

SigSNP = Significant SNP

Raw P = Raw p-value

Bonferroni adjusted P = p-values adjusted by Bonferroni correction (Raw P * N)

For the 100kb analysis, we identified significant eQTL in *IL5* and *IL6*. The *IL5* and *IL6* loci include haplotypes that were significantly associated with worm burden in Table 3.

**Fig 2:**
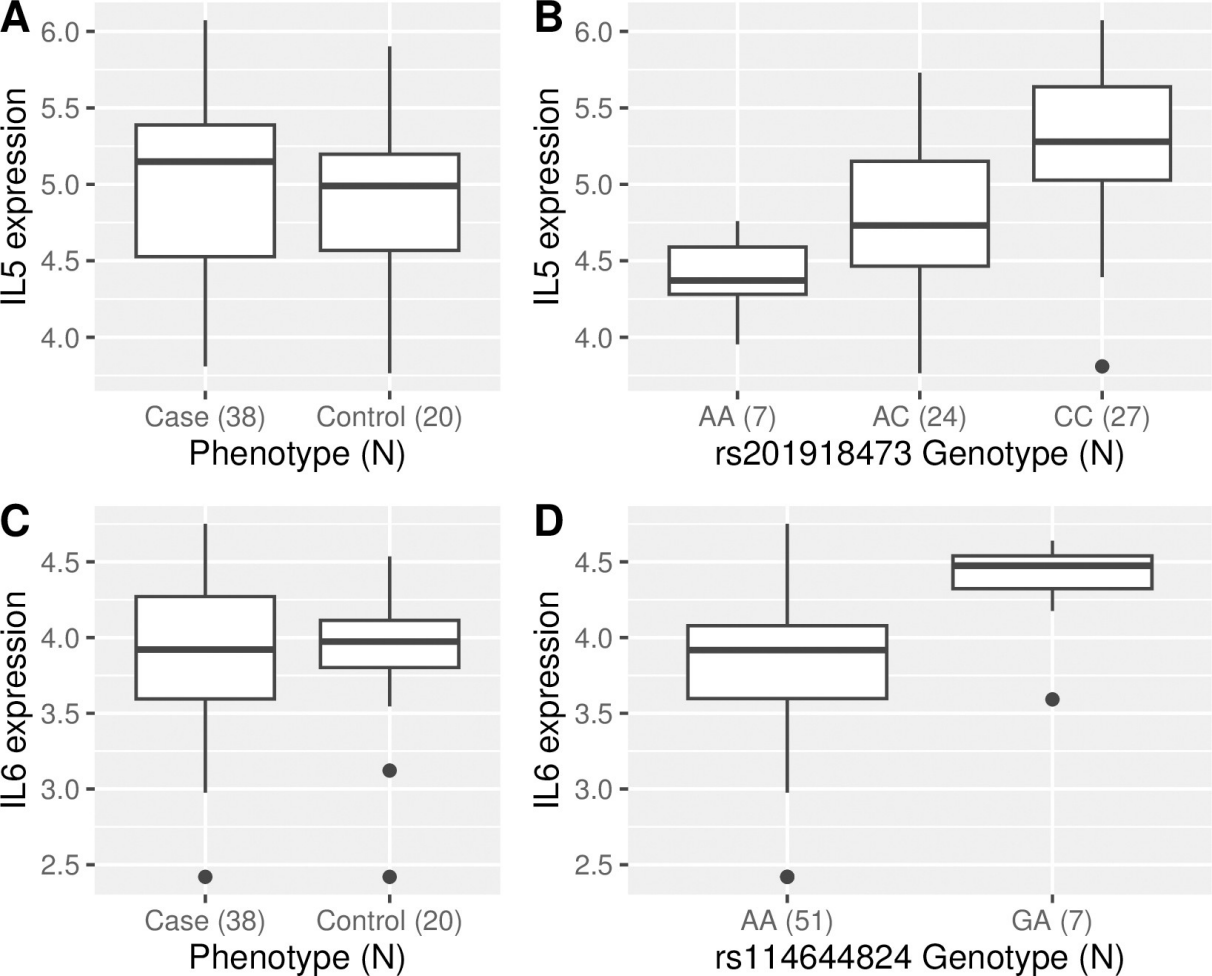
*IL5* and *IL6* expression by phenotype and genotype. Boxplot showing *IL5* and *IL6* expression by phenotype and genotype in the x-axis; while the y-axis is the normalized read count per gene on a log scale. In both cases there is a significant association with genotype but not with phenotype. **(**A) *IL5* expression by phenotype and (B) I*L5* expression by genotype of SNP rs201918473. (C) *IL6* expression by phenotype (D) *IL6* expression by rs114644824, there were no participants with a GG genotype at this SNP. Case = Those with CAA > = 30 pg/ml and Control = Samples with CAA<30 pg/ml [22,52].

In the 1Mb analysis, more significant eQTL loci were identified, including *IL25*, *IFNG* and *IL21*. In the 1Mb analysis, the *IL5* locus, which was significant in the 100Kb analysis, was no longer significant because the larger number of SNP tested led to a stronger correction for multiple testing, but shows a trend towards significance (p = 0.08). Although there were multiple SNP associated with expression of *IL5* these were not associated with worm burden as measured by UCP-LF CAA (Fig 2), indicating that the eQTL may be unrelated to schistosomiasis infection intensity.

## Discussion

We have tested 24 candidate genes for association with *S*. *mansoni* worm burden in children residing in the Albert Nile of Uganda. Approaches used included SNP, haplotype and eQTL analyses. We present results for SNP in Tables 2 and S2, and for haplotypes in Tables 3 and S3. 191 SNPs and 99 haplotypes show associations at the p < 0.05 level but did not remain significant after adjusting for multiple testing by Bonferroni correction (S2 and S3 Tables respectively). We used the Bonferroni correction with the number of haplotype blocks as the denominator as it can minimize false positive findings especially in genetic tests where multiple markers are tested simultaneously [53]. Bonferroni correction is recommended for hypothesis free tests but results in increased risk of false negatives which can result in missed associations [54]. Some SNPs may not pass Bonferroni correction but can still be replicated in multiple studies. For example, in a recent review we conducted for loci affecting worm burden [12], two SNPs (rs1800925 in *IL13* and rs2243250 in *IL4*) were replicated in multiple studies [25,28,29,55,56], despite the original studies reporting the loci without adjusting for multiple testing. The associations that we have found generally have odds ratios below the thresholds that our study was powered to detect. Therefore, there is need to replicate in future studies the loci that we report for their effect on worm burden and insights on host-worm biology.

Several top-ranking SNPs and haplotypes with p<0.05 are in the *IL6* locus. These SNPs were in regions of the genome that had elevated conservation and DNAse sensitivity scores (Fig 1) suggesting that they might be functional. We also found that SNP rs114644824 and log CAA were associated with *IL6* expression (Bonferroni unadjusted p<0.05), even though SNP rs114644824 was not itself associated with log CAA. The above evidence suggests that IL6 may play an important role in modulating worm burden. In vitro evidence has shown that *IL6* is expressed in cells of acute inflammatory granulomas induced by *S*. *mansoni* eggs in mice and that it enhances the cytotoxic activity of human platelets against *S*. *mansoni* larvae. This suggests that IL6 may play a role in the immune response to schistosome eggs and the development of egg-induced immunopathology such as liver granulomas during schistosomiasis [57,58]. A study in Sudan observed increased IL6 levels in *S*. *mansoni* infected individuals compared with non-infected individuals, and that levels correlated with egg load [58]. However, a recent study in Western Kenya has found there is no difference in IL6 plasma concentrations between infected and uninfected individuals, and there was no association with egg load [59]. Differences in associations with IL6 in the above studies may be partially attributable to the variability of Kato-Katz in accurately capturing infection intensity compared to more sensitive methods like UCP-LF CAA [22].

We also found that *IL10* contains SNPs and haplotypes with p<0.05. This association appeared to be mediated only by particular haplotypes of IL10 polymorphisms rather than the SNP in isolation, since in our results, the *IL10* promoter SNPs rs1800871, rs1800872 and rs1800896 were not individually associated with worm burden despite the haplotype GC of these SNP being associated with CAA levels (Table 4). This is in keeping with the mixed results from studies of the associations of these SNPs with infection intensity [26,27,29]. Further evidence of *IL10* association is from a Cameroonian family-based study [60] where the rs1800871C was associated with low worm burdens while rs1800872A was associated with high worm burdens. IL6 and IL10 cytokines have contrasting immune effects, IL6 is primarily proinflammatory, and it is possible that to control the effect of inflammation the host produces the anti-inflammatory IL10. Another possible explanation for elevated IL10 levels may be co-infection with other chronic parasitic infections, since plasma IL10 levels were found to be elevated in *Schistosoma haematobium* coinfection with malaria [61]. Coinfection data was not available for the current analysis.

Further, through eQTL analysis, we have showed multiple significant SNPs modulating *IL5* expression in our dataset. The *IL5* gene is contained in the classical *S*. *mansoni* susceptibility locus SM1, that was the first locus to be causally associated with *S*. *mansoni* worm burden [11]. These eQTL SNPs remained significant even after Bonferroni correction, suggesting that they are differentially expressed in high versus low worm burden. We also found additional SNPs that are significantly associated with expression *of IFNG*, *IL21* and *IL25* genes. Although we did not find direct genetic polymorphisms linking polymorphisms in *IFNG* to worm burden, it’s receptor, the *(IFN-gammaR1) on chromosome 6q22-q23* is part of the second locus to be associated with *S*. *mansoni* outcomes, termed SM2 locus [62] which was primarily associated with severe hepatic fibrosis. IFNG has also been associated with increased time to re-infection among frequently exposed car washers in Western Kenya [25]. It is possible that IFNG is involved in regulating both fibrotic pathological processes and resistance to increased worm burden. To our knowledge, *IL25* and *IL21* polymorphisms in the Th17 pathway have not been previously associated with worm burden but we have recently hypothesized that they might play a role in worm infection response as they are Th17 pathway genes within SM1 and SM2 loci [12,20]. Th17 responses have been shown to be important in the formation of granulomas and the pathology of schistosomiasis [12,63–65]. There were elevated Th17 responses in egg-induced hepatic pathology in murine infection with *S*. *japonicum* [63], while neutralization of Th17 responses significantly ameliorated hepatic granulomatous inflammation and liver damage in *S*. *japonicum*-infected mice [65]. There is evidence that the protective role of Th17 cytokines (IL17 and IL22) against *S*.*mansoni* soluble egg antigen-induced granuloma formation is through regulating granulocyte recruitment and functions [64]. The evidence for a role for Th17 genes in the control of worm burden is weaker, however decreasing IL17 with an anti-IL17 antibody provided partial protection against *S*. *japonicum* infection in mice [66].Other genes in the Th17 pathway that were suggestive for associations with worm burdens were *IL17B* and *IL12B*, which were significant in SNP and haplotype analysis. Further published evidence of Th17 pathway involvement in schistosomiasis include elevation of IL17 in a murine immunopathology model [67] and elevation of Th17 cells in children infected with *S*. *haematobium* compared to those that are uninfected [68].

Another suggestive worm burden association was with *RNASE3*, also known as *ECP*, which is a secretory protein of eosinophil granulocytes that efficiently kills the larval stage of *S*. *mansoni*. *RNASE3* has been associated with schistosomiasis infection, infection intensity and fibrosis in a study that was conducted among the Alur speaking community of Lake Albert [34]. Notably this is a similar ethnic population and geographical area as the current study, and therefore our findings replicate this prior study.

Comparing several studies for replication of SNP effects is challenging since different SNP may regulate a particular response in different populations [69]. This phenomenon, termed genetic heterogeneity, therefore points to effects more at a gene level or combining several SNPs as the way forward. We have found effects at haplotype level which were not shown at SNP level. In our analysis, we test haplotypes within 100 kilobases of each gene, and these loci may contain multiple genes. Any of the genes in these loci could be the effector gene that is impacted by SNP or haplotype and therefore these require functional studies to validate the effects.

Strengths of the current study include the design to capture differences due to worm burden at the three levels of phenotyping, genotyping and analysis. For the phenotyping, we used the most sensitive diagnostic detecting worm-derived circulating antigen, UCP-LF CAA which is more sensitive than KK, the current WHO gold standard [4,22]. In genotyping we used a H3Africa pan-African genotyping chip customized for African genomes and therefore is expected to have higher power to detect associations in African populations than chips designed primarily with data from Western-European participants. Our analysis approach where we used extreme phenotype sampling increases power and is cost effective. We also used haplotypes to increase power and linear models to allow us to incorporate covariates in our analyses.

However, there are some limitations, such as insufficient sample size for whole genome analysis. We have mitigated this by focusing only on candidate genes and conducting extreme phenotype sampling to maximize the power to detect effects. None of the markers for SNP and haplotype analysis were significant post-Bonferroni, but eQTL markers in 5 genes(I*L5*,*IL6*, *IFNG*,*IL21* and *IL25*) were significant. Bonferroni correction may also have resulted in overcorrection, missing actual genetic markers associated with worm burden. The markers presented here represent hypotheses that could be tested in additional studies to confirm their associations with worm burden. It is also likely that more SNPs control worm burden than those analyzed. The results presented are also from one timepoint in a cross-sectional study. Longitudinal studies are more powerful to capture time course and re-infection events which may generate causal evidence of the effect of immune markers [70]. Another approach that may validate the genes in the current study is controlled human infection trials [71], where the worm burden introduced will be known and it will be possible to compare gene expression and immune markers before infection and after infection. We also recommend the standard validation approach of conducting replication studies which find the same signals as the current study. Another approach is to link the genetic markers identified with the functional role of the genes in schistosomiasis worm burden and pathology in the literature, as we have done in the current study.

Ideally, our candidate gene analysis and eQTL analysis should identify the same SNP associated with both gene expression and worm burden. In our analysis, different SNPs were associated with either of the traits, possibly due to our analysis of blood, a heterogenous tissue consisting a mix of cell types, some of which are more related to the phenotype of worm burden than others. It is therefore possible that responses to worm burden are regulated via changes in gene expression, but the mix of cell types we assayed in blood may confound detection. The approach we used to mitigate the differences between eQTL analysis and candidate SNP analysis is to compare the result of both approaches to identify overlapping variants or genes. In our data, *IL6* appeared in both SNP, haplotype and eQTL analyses, increasing our confidence in the association with worm burden.

It is also possible that we did not capture strictly *S*. *mansoni* worm burden, as hybrid forms have been documented before [72], and the worm burden measured by CAA is not species specific [73]. There is evidence that the CAA based diagnostic test detect hybrids in humans[74] and in mice monoclonal antibodies directed against CAA were produced from hybridomas derived from schistosome-infected mice or mice immunized with antigen preparations of different schistosome species [75]. Therefore, our results are interpreted as *S*.*mansoni* worm burden schistosomiasis based on the most prevalent worm present known to be in the region. The hybrid schistosomiasis, CAA detection and genetic susceptibility remain an area for further studies.

The practicality of host genetic testing during mass drug administration is an issue that needs careful consideration. A set of just 26 genetic markers can explain 45% of the variance of schistosomiasis associated hepatic fibrosis in a Brazil population [76]. A similar set of markers that could identify those at high genetic risk of having high worm burdens combined with epidemiological data could assist in the targeting of mass drug administration. The WHO 2006 guidelines targeted MDA at school age children because they often have the heaviest worm burdens and are most easily targeted for treatment. However, the 2022 update to the MDA guidelines has widened the scope of MDA to younger children and adults [77]. Now that it is envisioned that individuals might need regular treatment for a decade or more the savings that could be achieved by targeting treatment at those with highest worm burdens might outweigh the additional cost and complexity of screening and genotyping individuals at an early stage in the program. A South African study estimated the cost of MDA at about $14 per treatment in 2012 [78]. Treating 100 people annually for a decade would cost about $14,000. If the 20 or 30 of people out of the 100 with the heaviest worm burdens and highest risk could be targeted that could save around $10,000. Therefore, if the cost of screening and genotyping individuals was less than $100 a net saving could be achieved. Since genotyping 30 loci can cost less than $10 such a policy should be practicable if a suitable panel of genotypes can be identified. There would also be need for easy tools for genotyping that can be deployed in the field, which can be developed once a suitable panel of susceptibility markers is identified.

## Conclusion and recommendations

Individuals with polymorphisms in loci containing *IL6*, *IL10*, *FCN2*, *RNASE3* and multiple Th17 pathway genes such as *IL12B* and *IL17B* had varying worm burden, indicating that these loci may play a role in determining infection intensity. To develop novel therapies, there is need to conduct functional studies that can elucidate the roles of the genes in the loci in controlling worm burden. If robust associations with worm burdens are proven in multiple populations, these polymorphisms can be used as biomarkers for targeting control efforts to individuals most likely to carry high worm burdens and continually shed eggs into the community. We hope this will ultimately lead to higher efficiency of control programs to reduce schistosomiasis worm burdens in endemic populations.

## Supporting information

S1 Table SNP and Individual quality control filters of whole genome data. (XLSX)

S2 Table Full results of Candidate gene SNPs association with CAA worm burden. (XLSX)

S3 Table Candidate gene haplotypes associated with CAA worm burden(p<0.05). (XLSX)

S4 Table Candidate gene haplotypes (p<0.05) containing SNPs (p<0.05) associated with CAA worm burden. (XLSX)

S5 Table Candidate gene haplotypes(p<0.05) containing published SNPs investigated for egg/worm burden. (XLSX)

S6 Table Candidate gene SNP identified in literature for association with worm burden and annotated with variant effect predictor. (XLSX)

S1 Appendix List of unique BigLD haplotype blocks and FDR of top alleles per block within 100kb of candidate genes. (XLSX)

S1 Fig Study design summarizing methods from sample collection to analysis. (TIF)
