# Supplementary figures and images for: Variants of IL6, IL10, FCN2, RNASE3, IL12B and IL17B loci are associated with Schistosoma mansoni worm burden in the Albert Nile region of Uganda
Source: PLoS Negl Trop Dis. 2023 Nov 30;17(11):e0011796. doi: 10.1371/journal.pntd.0011796 (PMC10715658; doi:10.1371/journal.pntd.0011796)

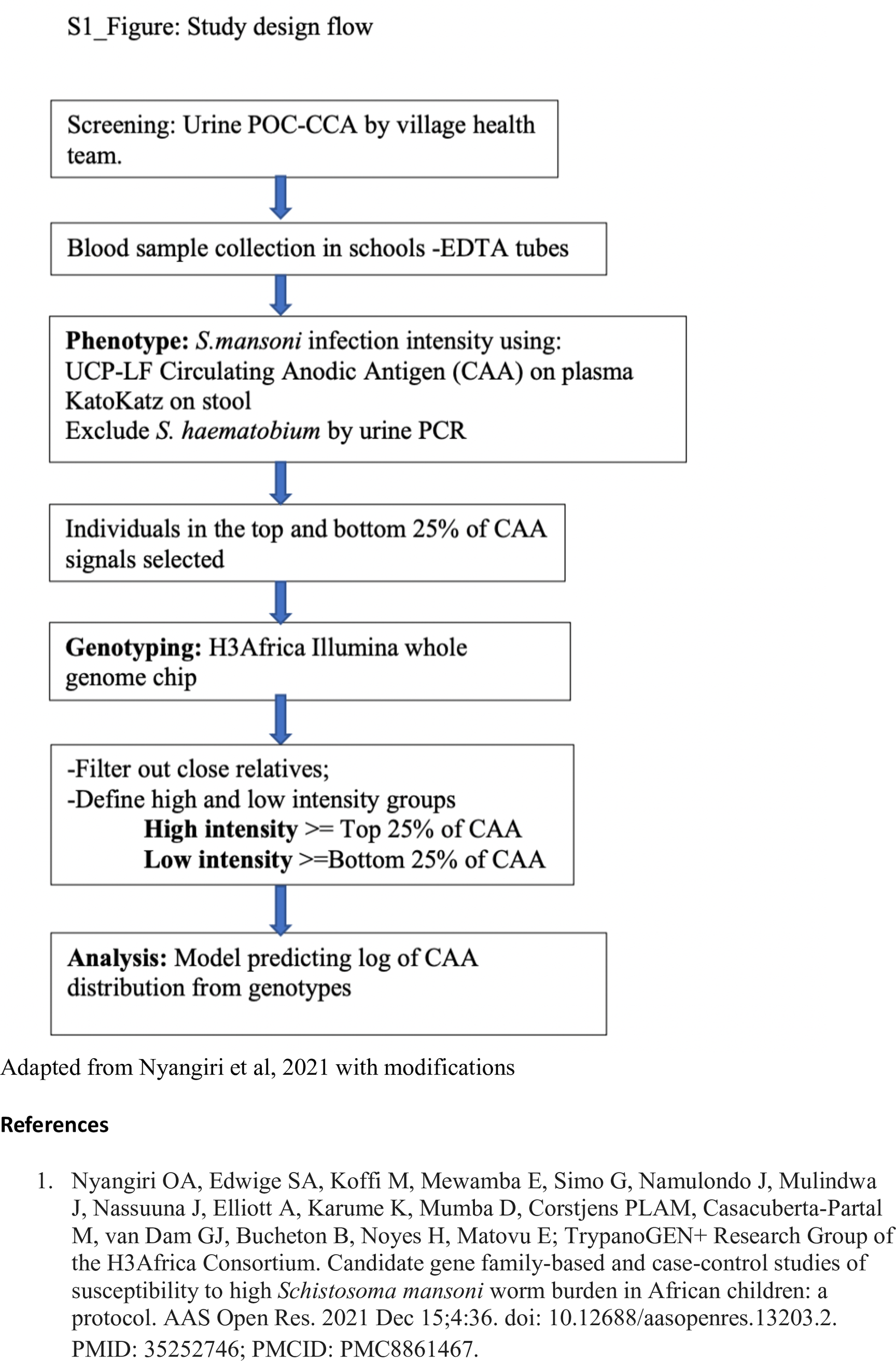

Supplement: S1 Fig — (TIF) [file pntd.0011796.s008.tif]
